# Cirrhosis is not a contraindication to cytoreductive surgery and hyperthermic intraperitoneal chemotherapy in highly selected patients

**DOI:** 10.1186/s12957-018-1389-3

**Published:** 2018-04-26

**Authors:** Anna Weiss, Erin P. Ward, Joel M. Baumgartner, Andrew M. Lowy, Kaitlyn J. Kelly

**Affiliations:** Department of Surgery, Division of Surgical Oncology, University of California, San Diego, Moores Cancer Center, 3855 Health Sciences Dr. Mail Code 0987, La Jolla, CA 92093 USA

**Keywords:** Cytoreductive surgery, Cytoreduction, HIPEC, Cirrhosis

## Abstract

**Background:**

Patient selection for cytoreductive surgery (CRS) and hyperthermic intraperitoneal chemotherapy (HIPEC) is critically important to optimizing outcomes. There is currently no literature regarding the safety of CRS/HIPEC in patients with cirrhosis. The aim of this case series is to report the outcomes of three patients with well-compensated cirrhosis who underwent CRS/HIPEC.

**Methods:**

Patients were identified from a prospectively maintained peritoneal surface malignancy database. Patient, tumor, and operative-related details were recorded as short-term postoperative outcomes. Results were analyzed using descriptive statistics.

**Results:**

All patients had well-compensated (Child-Pugh Class A) cirrhosis and Eastern Cooperative Oncology Group (ECOG) performance status of 0. One patient had preoperative evidence of portal hypertension. All safely underwent CRS/HIPEC with completeness of cytoreduction (CC) scores of 0. The postoperative morbidity profile was unique, but all complications were manageable and resulted in full recovery to preoperative baseline status.

**Conclusions:**

Patient selection for CRS/HIPEC is critical for optimization of short- and long-term outcomes. This small series suggests that well-compensated cirrhosis should not be an absolute contraindication to CRS/HIPEC.

## Background

Cytoreductive surgery (CRS) and hyperthermic intraperitoneal chemotherapy (HIPEC) is now widely accepted as the standard of care for peritoneal surface malignancy (PSM) from appendiceal, colorectal, and primary peritoneal malignancies. Cytoreductive surgery alone may carry a high risk of morbidity even without considering the addition of HIPEC. Postoperative morbidity rates for CRS/HIPEC are reported between 40 and 52%, and major morbidity at 17–24% [1–8]. In-hospital mortality varies from 0 to 4.4% and 30-day mortality from 2.4 to 8% [1–4, 9]. Reoperation rates are as high as 15–17.5%, and length of stay has been reported to be between 8 and 16 days on average. [1–3, 9, 10]

Multiple studies have evaluated outcomes of non-hepatic surgery in patients with cirrhosis [11]. In general, rates of postoperative morbidity and mortality are higher in cirrhotic patients following major intraabdominal surgeries than for non-cirrhotic patients undergoing similar procedures. For example, El Nakeeb and colleagues reported statistically significantly higher rates of wound complications, intraabdominal hemorrhage, pancreatic fistula, and hospital mortality in cirrhotic patients than non-cirrhotics following pancreaticoduodenectomy [12]. The authors concluded that the operation should only be performed in patients with well-compensated cirrhosis—those with Child-Pugh class A disease, and no portal hypertension. In a series of 772 patients with cirrhosis undergoing major surgery, several factors affected outcome—age, American Society of Anesthesiology (ASA) class, and model for end-stage liver disease (MELD) score were found to predict mortality at 30 and 90 days postoperatively, independent of the procedure performed [13]. Risks of elective major surgery were low in patients with a MELD score of less than 11. Risks were so high in those with a MELD score of ≥ 20; it was recommended that all elective procedures be postponed until after liver transplantation. In patients with an intermediate MELD score of 12 to 19, it was recommended that evaluation for transplantation be completed prior to elective major surgery so that transplant could be expedited postoperatively if required. [13]

CRS/HIPEC has never been examined in patients with cirrhosis. While patients with cirrhosis are known to have increased risk of morbidity and mortality following laparotomy, there are theoretically no explicit contraindications to CRS/HIPEC, and hepatotoxicity related to intraperitoneal (IP) agents has not been reported. The aim of this report is to describe a small series of patients with Child-Pugh class A cirrhosis who safely underwent CRS/HIPEC.

## Methods

### Patients

A retrospective analysis of a prospectively maintained peritoneal surface malignancy database from a single, high-volume CRS/HIPEC center was completed. It was noted that within the past 2 years, several patients with known cirrhosis presented for evaluation for CRS/HIPEC. Three individuals with cirrhosis who underwent CRS/HIPEC were identified in the Research Electronic Data Capture (REDCap) software-based database and reviewed. This prospectively maintained REDCap database is maintained in accordance with our institutional review board.

### Data

Preoperative, operative, and postoperative data for all three patients was evaluated. Preoperative variables included general demographics, past medical history, histologic diagnosis, Eastern Cooperative Oncology Group (ECOG) performance status, body mass index (BMI), pre-operative symptoms, etiology of cirrhosis, laboratory values, Child-Pugh score, MELD score, and prior treatment for cirrhosis. Operative variables collected included peritoneal carcinomatosis index (PCI), cytoreductive procedures performed, operative time, estimated blood loss (EBL), urine output, intraoperative transfusion, HIPEC agent, and completeness of cytoreduction (CC) score. Postoperative variables collected included intensive care unit (ICU) length of stay (LOS) and total hospital admission LOS, time to return of bowel function defined as first flatus, postoperative transfusion, 30-day morbidity, serum bilirubin at discharge, international normalized ratio (INR) at discharge, and platelet count at discharge. Complications were classified per the Clavien system [14]. Patient-specific data and outcomes were analyzed with descriptive statistics.

### Surgery

In all cases, a laparoscopy was performed prior to the definitive procedure for assessment of disease burden and candidacy for definitive CRS/HIPEC without associated complications. At time of laparotomy, the peritoneal surfaces were systematically inspected and the small bowel was examined from the ligament of Treitz to the ileocecal valve. The PCI as defined by Sugarbaker was determined, and assessment of potential complete cytoreduction (CC 0 or 1) and removal of all gross disease was completed [15]. CRS/HIPEC was performed utilizing our standardized methods as previously described [16]. The intraperitoneal chemotherapy solution was heated to 42 °C and infused for 90 min, as is the standard approach. This was not changed for these cirrhotic patients.

## Results

Two Caucasian male patients and one Hispanic female were identified. The median age of the patients was 49 years (range 44 to 59), and patient BMI ranged from 24 to 37 kg/m^2^ (median 32). The two male patients presented with low-grade appendiceal mucinous neoplasm (LAMN) and the female patient presented with peritoneal mesothelioma. Two patients denied symptoms at presentation, and one presented with isolated right lower quadrant abdominal pain. Both asymptomatic patients were initially diagnosed with peritoneal pathology found incidentally on cross-sectional imaging completed for liver disease. All patients underwent a baseline laparoscopy prior to CRS/HIPEC for assessment of disease burden prior to laparotomy. One patient had the initial assessment during a laparoscopic right hemicolectomy at a referring institution. The etiology of cirrhosis included hepatitis C for two patients and alcohol for the other. All patients had normal hepatic function at baseline (Child-Pugh Class A), one had ascites at presentation, and one had sequelae of portal hypertension. Demographic and baseline clinical characteristics are summarized in Table 1.

**Table 1:** Preoperative patient and tumor-related variables

	Patient 1	Patient 2	Patient 3
Age (years)	49	59	44
Gender	M	M	F
Disease type	LAMN	LAMN with focal high grade	Malignant mesothelioma
ECOG	0	0	0
ASA class	III	III	IV
BMI (kg/m^2^)	37	24	32
Symptoms	None	None (right lower quadrant pain prompted laparoscopic appendectomy turned right hemicolectomy)	Weight loss, abdominal distension, lower extremity edema
Etiology of cirrhosis	HCV	Alcohol	HCV cirrhosis, GT1a
Bilirubin	0.5	0.51	0.3
INR	1.2	1.1	1.0
Ascites (yes/no)	No	No	Yes
Child’s class	A	A	A
Platelet count	164	207	259
Portal hypertension	No	Yes	No
MELD score	8	8	6
Treatment for hepatitis C/cirrhosis	None	Alcohol cessation, propranolol for gastric varices	None
Other significant PMH	HTN, non-insulin-dependent diabetes	None	Insulin-dependent diabetes

*ECOG* Eastern Cooperative Oncology Group, *ASA* American Society of Anesthesiology, *BMI* body mass index, *INR* international normalized ratio, *MELD* model for end-stage liver disease, *PMH* past medical history

All patients underwent open CRS/HIPEC with complete clearance of all gross disease (CC 0). The median PCI was 9 (range 6 to 12). Procedures performed included omentectomy, selective peritonectomy including liver surface, excision of bowel nodules with serosal repair, ileocecectomy, hysterectomy, and oophorectomy. Operative time ranged from 306 to 463 min (median 368). The median estimated blood loss was 200 mL (range 200 to 500) and median urine output was 675 mL (range 500 to 880). One patient received an intraoperative blood transfusion (2 units). HIPEC was performed with mitomycin C (30 to 40 mg) for the patients with LAMN, and with cisplatin (182 mg) and doxorubicin (27 mg) for the patient with peritoneal mesothelioma. Operative data are summarized in Table 2.

**Table 2:** Operative variables

	Patient 1	Patient 2	Patient 3
Date	2/16/2011	2/17/2015	7/10/2015
PCI	12	6	9
Epidural (yes/no)	No	No	Yes
Procedures performed	Excision of tumors on sigmoid colon and cecum, pelvic peritonectomy, liver wedge biopsy, omentectomy	Omentectomy, selective peritonectomy, ventral hernia repair	Diagnostic laparoscopy, Omentectomy, extensive peritonectomy, including bilateral diaphragms, ileocecectomy with primary anastomosis, hysterectomy, left oophorectomy
Operative time (min)	306	463	368
Estimated blood loss (cm^3^)	200	200	500
Urine output (cm^3^)	500	675	880
Need for transfusion	No	No	Yes (2 units PRBC, 1 unit FFP)
HIPEC agent	30 mg mitomycin C	40 mg mitomycin C	182 mg cisplatin, 27 mg doxorubicin
CC score	0	0	0

*PCI* Peritoneal Cancer Index, *CC* completeness of cytoreduction, *HIPEC* hyperthermic intraperitoneal chemotherapy

Postoperatively, the median length of ICU and overall hospital stay were 1 and 13 days, respectively. The median time to return of bowel function was 4 days (range 4 to 6). All patients experienced a postoperative morbidity within 30 days. Two patients who did not have ascites preoperatively developed symptomatic ascites in the postoperative period. These patients were managed with paracentesis, spironolactone, and sodium restriction. In one case, the ascites fluid became infected and treatment with IV antibiotics was required. Additional complications included urinary tract infection, ileus, and one superficial wound infection. No patients developed laboratory evidence of hepatic insufficiency postoperatively, and no patients developed long-term sequelae (repeated paracentesis after discharge, for example) of hepatic insufficiency. Postoperative data are summarized in Table 3. The two patients with LAMN are both clinically No Evidence of Disease (NED) at 6 and 46 months from the date of CRS/HIPEC. The patient with peritoneal mesothelioma remains clinically NED at 15 months following CRS/HIPEC.

**Table 3:** Postoperative variables

	Patient 1	Patient 2	Patient 3
ICU length of stay (days)	1	1	2
Length of stay (days)	22	7	13
Time to NG or g-tube removal (days)	None placed	None placed	2
Time to first flatus (days)	4	4	6
Need for postoperative blood transfusion	None	None	None
30-day morbidity/grade^a^	UTI/II Ascites/II SBP/II Ileus/II	Ascites/II	Superficial site infection/I
Bilirubin on discharge	1.1	0.63	0.75
INR on discharge	1.4	1.5	1.1
Platelet count on discharge	76	132	430
Final pathology	Acellular mucin, liver biopsy—cirrhosis	LAMN, acellular mucin	Epithelioid type mesothelioma

*ICU* intensive care unit, *INR* international normalized ratio

^a^Clavien-Dindo classification

## Discussion

CRS/HIPEC carries significant morbidity, but despite the known risks, it remains the standard of care for peritoneal surface malignancy including appendiceal, colorectal, and primary peritoneal tumors [15, 17–19]. CRS/HIPEC allows for a uniquely effective treatment for diseases that otherwise have limited safe, systemic-based chemotherapy options. The use of direct IP administration of chemotherapy during HIPEC allows for markedly higher drug delivery to peritoneal-based tumors than can be safely achieved with systemic administration [10]. The IP administration allows patients to gain the benefits of high drug concentration while minimizing the risk of systemic toxicity associated with the commonly used drugs. Currently, the most common side effects of IP administration of HIPEC agents at the time of CRS include ileus, acute kidney injury, neutropenia, impaired wound healing, and anastomotic dehiscence [7, 20].

As careful patient selection for CRS/HIPEC is critical to optimize outcomes, recent research efforts have investigated the safety of CRS/HIPEC in specific patient populations, such as the elderly and the morbidly obese [16, 21–23]. These studies have served to better define which patients are eligible for treatment, and to allow for more informed discussions about the expected postoperative course in these specific patient populations. Alyami and colleagues reported that patients greater than 70 years of age undergoing CRS/HIPEC had significantly more cardiovascular complications than younger patients, but had no significant difference in 90-day overall morbidity or mortality [21]. Obesity has been shown to be associated with increased risk of renal, pulmonary, and wound complications following CRS/HIPEC, but has not been associated with serious morbidity or mortality [16, 22]. Several groups have concluded that obesity should not even be a relative contraindication to treatment [24]. To date, there have been no previous reports of CRS/HIPEC being performed in patients with cirrhosis, a growing subset of the population with a unique comorbidity profile [11].

**Fig. 1:**
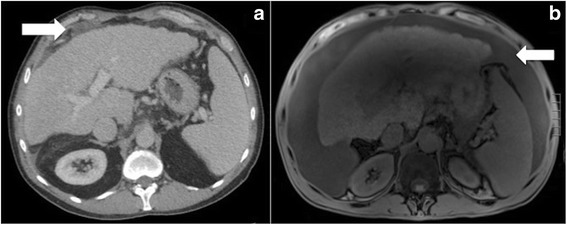
**a** Preoperative computed tomography scan of a patient with pseudomyxoma peritonei from low-grade appendiceal mucinous neoplasm with cirrhosis. Arrow shows mucinous implants on the peritoneal surface of the right hemi-diaphragm. **b** Magnetic resonance imaging scan at 6 months postoperatively showing diffuse ascites. This fluid was removed by paracentesis and was serous. Cytology was negative for mucin or malignant cells, and the ascites was resolved with sodium restriction and diuresis

In the current case series, we identified three cirrhotic patients that were successfully treated with CRS/HIPEC, although not without some complication. Two of the three patients who did not have ascites preoperatively developed serous ascites in the postoperative setting. Both cases were successfully managed with diuresis and sodium restriction. In contrast, the patient with peritoneal mesothelioma that did have serous ascites preoperatively had complete resolution of ascites after CRS/HIPEC. This patient’s paracentesis preoperatively showed “atypical mesothelial cell proliferation,” suggesting that the fluid was related to malignancy and not to underlying liver disease. These findings suggest that serous ascites in patients presenting with cirrhosis and PSM may be secondary to either etiology. Cirrhosis-related ascites significantly impacts Child’s score and perceived operative risk and some consider ascites a contraindication to CRS/HIPEC in PSM of colorectal origin; however, ascites may be the result of the patient’s peritoneal malignancy presenting a unique diagnostic quandary. Our findings support performing paracentesis with analysis of fluid for electrolytes and protein, and cytology should be considered to determine the etiology of ascites prior to excluding patients based solely on the presence of ascites. Similarly, postoperative ascites may not reflect recurrent disease but may be cirrhosis related. This was the case for one of the patients in this study with LAMN. Preoperatively, the patient had mucinous implants on his peritoneum and omental thickening but no ascites. Six months postoperatively, he developed serous ascites (Fig. 1a, b), but paracentesis revealed fluid cytology without mucin or malignant cells and his ascites ultimately resolved with sodium restriction and diuresis.

Many features of CRS/HIPEC make it unique relative to other major abdominal surgeries. These include the administration of hyperthermia and IP chemotherapy, the likelihood of prolonged operative time and accompanying significant fluid shifts. Patients in this study received either MMC or cisplatin/doxorubicin as HIPEC agents. MMC is a cytotoxic antibiotic derived from fungus that acts by cross-linking DNA and has wide anti-tumor activity. The most common systemic toxicities are myelosuppression, nephrotoxicity, and pulmonary fibrosis. Cisplatin is a platinum-based compound, and the most common systemic toxicities associated with cisplatin are nephrotoxicity, mild peripheral neuritis, nausea and vomiting, and ototoxicity [25]. Doxorubicin is cleared predominantly by the liver, and the most common systemic toxicities are myelotoxicity, mucositis, alopecia, and cardiomyopathy [25]. None of these agents are associated with hepatotoxicity with systemic administration. It is therefore unlikely that they would cause hepatotoxicity with IP administration.

One factor that could theoretically lead to liver dysfunction in cirrhotic patients undergoing CRS/HIPEC is prolonged operative time and prolonged exposure to general anesthesia. In this series, the longest procedure was 463 min. CRS/HIPEC cases for patients with extensive peritoneal disease burden can be in excess of 10 to 12 h. It is likely that such extended operative time would add significant risk for patients with cirrhosis, even if well compensated.

A significant limitation of this report is the small number of patients. However, the primary aim of this review was not to say that all patients with well-compensated cirrhosis can safely undergo CRS/HIPEC. Rather, our results demonstrate that CRS/HIPEC can be safely undertaken in select patients with well-compensated cirrhosis and describes the baseline and operative characteristics of three patients that were successfully treated. As patients with metastatic cancer are not candidates for liver transplantation, decisions about undergoing major surgery need to be made very carefully in this population.

We recommend extrapolating previous recommendations for elective major surgery in cirrhotic patients to CRS/HIPEC. In the current study, all patients had a MELD score < 11, which has been shown in a large retrospective review of major gastrointestinal, orthopedic, and cardiovascular operations, to portend a 10% surgical mortality risk [13]. Considering the nuances of CRS/HIPEC specifically, we recommend consideration of the estimated operative time, disease burden, and overall cancer prognosis in determining if patients with cirrhosis are candidates for surgery. Patients with LAMN are able to achieve long-term survival following complete cytoreduction and HIPEC with CC 0 or 1 cytoreduction [1, 26]. Those with colorectal cancer or peritoneal mesothelioma, however, should only be selected if they have limited disease burden and CC 0 cytoreduction can be achieved [27]. Additionally, patients presenting with ascites should have fluid studies to determine if it is related to malignancy or liver disease. We recommend selection of non-hepatotoxic drugs for IP therapy in cirrhotic patients and early hepatology consultation for perioperative management.

## Conclusions

Patient selection for CRS/HIPEC is critical for optimization of short- and long-term outcomes for all patients. This report will hopefully provide some initial framework for clinicians encountering patients with cirrhosis and PSM and motivate others to similarly report their experience with this challenging patient population to allow for larger studies to be performed in the future.

## Abbreviations

ASA: American Society of Anesthesiology
BMI: Body mass index
CC: Completeness of cytoreduction
CRS: Cytoreductive surgery
EBL: Estimated blood loss
ECOG: Eastern Cooperative Oncology Group
HIPEC: Hyperthermic intraperitoneal chemotherapy
ICU: Intensive care unit
INR: International normalized ratio
IP: Intraperitoneal
LAMN: Low-grade appendiceal mucinous neoplasm
LOS: Length of stay
MELD: Model for end-stage liver disease
NED: No Evidence of Disease
PCI: Peritoneal carcinomatosis index
PSM: Peritoneal surface malignancy
REDCap: Research Electronic Data Capture
